# Effects of Phytic Acid-Degrading Bacteria on Mineral Element Content in Mice

**DOI:** 10.3389/fmicb.2021.753195

**Published:** 2021-11-22

**Authors:** Diao Zhou, Ying Zhao, Jing Li, Vinothkannan Ravichandran, Leli Wang, Qiuyun Huang, Cang Chen, Hengjia Ni, Jia Yin

**Affiliations:** ^1^Key Laboratory of Protein Chemistry and Developmental Biology of Fish of Ministry of Education, Hunan Provincial Key Laboratory of Animal Intestinal Function and Regulation, Hunan International Joint Laboratory of Animal Intestinal Ecology and Health, Hunan Normal University, Changsha, China; ^2^Key Laboratory of Agro-Ecological Processes in Subtropical Region, Hunan Provincial Key Laboratory of Animal Nutritional Physiology and Metabolic Process, Hunan Research Center of Livestock and Poultry Sciences, South Central Experimental Station of Animal Nutrition and Feed Science in the Ministry of Agriculture, Institute of Subtropical Agriculture, Chinese Academy of Sciences, Changsha, China; ^3^State Key Laboratory of Microbial Technology, Shandong University-Helmholtz Institute of Biotechnology, Shandong University, Qingdao, China

**Keywords:** *Lactococcus lactis* psm16 strain, phytic acid, phytase, short-chain fatty acid, trace minerals

## Abstract

Trace minerals are extremely important for balanced nutrition, growth, and development in animals and humans. Phytic acid chelation promotes the use of probiotics in nutrition. The phytic acid-degrading strain *Lactococcus lactis* psm16 was obtained from swine milk by enrichment culture and direct plate methods. In this study, we evaluated the effect of the strain psm16 on mineral element content in a mouse model. Mice were divided into four groups: basal diet, 1% phytic acid, 1% phytic acid + psm16, 1% phytic acid + 500 U/kg commercial phytase. Concentrations of acetic acid, propionic acid, butyric acid, and total short-chain fatty acids were significantly increased in the strain psm16 group compared to the phytic acid group. The concentrations of copper (*p* = 0.021) and zinc (*p* = 0.017) in liver, calcium (*p* = 0.000), manganese (*p* = 0.000), and zinc (*p* = 0.000) in plasma and manganese (*p* = 0.010) and zinc (*p* = 0.022) in kidney were significantly increased in psm16 group, while copper (*p* = 0.007) and magnesium (*p* = 0.001) were significantly reduced. In conclusion, the addition of phytic acid-degrading bacteria psm16 into a diet including phytic acid can affect the content of trace elements in the liver, kidney, and plasma of mice, counteracting the harmful effects of phytic acid.

## Introduction

Trace elements play an important role in the growth, development, and metabolism of animals and humans. Significant fluctuations in their levels can lead to metabolic disorders and immune dysfunction (Cannas et al., 2020). Phytic acid is present in many plant organs, accounting for 1–5% of most seeds, roots, and stems of grains and vegetables (Wang et al., 2013). Numerous animals feed on fiber-rich grains while the daily intake of humans is influenced by culture and society (Wang and Guo, 2021). Grains and vegetables are an important source of all nutrients, including minerals (Olza et al., 2017). Phytic acid is a sugar phosphate with six strong negative charges and has commonly been reported as an anti-nutritional factor in humans and animals due to its strong chelating ability (Kumar et al., 2021). Phytic acid inhibits the absorption of calcium, magnesium, zinc, iron, and other trace elements in humans and animals (Shi et al., 2018). Furthermore, phytic acid can also combine with cationic groups on food proteins, digestive enzymes, and lipids to form insoluble complexes in the gastrointestinal tract (Kumar et al., 2010). Endogenously secreted minerals cannot be absorbed and reabsorbed due to the lack of intestinal phytase, causing potential digestion problems in monogastric animals and humans (Hui et al., 2016).

Many animals feed on fiber-rich grains, while the daily human diet is influenced by culture and society (Wang and Guo, 2021). In recent years, phytate hydrolysis has attracted great attention as a mechanism to further improve the bioavailability of minerals and proteins. Phytases are enzymes capable of hydrolyzing phytic acid into phosphate and inositol with penta- to mono-phosphate intermediates and are widely distributed in nature in plants, animal tissues, and microorganisms (Rosa-Sibakov et al., 2018). Phytases in plant and animal tissues have minimal activity and weak stability, but microorganisms with phytase activity are highly effective in the promotion of mineral absorption in animals (Haros et al., 2001). Previous studies have indicated that the microorganisms *Aspergillus ficuum* (Zhang et al., 2010), *Aspergillus niger* (Corrêa and de Araújo, 2020), yeast (Greppi et al., 2015), and *Escherichia coli* are primary producers of phytase (Mezeli et al., 2017).

The beneficial properties of microorganisms depend mainly on the source of the bacterial strain (Lyons et al., 2020; Ren et al., 2021). Most microorganisms that produce phytase are isolated from the soil, rarely from breast milk. The latter provides nutrition and contains biologically active ingredients that guide the development of a newborn’s intestinal immune system (Cheng et al., 2021). Moreover, the maternal intake of phytic acid-rich foods may affect the bioavailability of mineral elements in breast milk. An iron, zinc, or calcium deficiency could lead to a profound adverse impact on the growth, health, and cognitive development of an infant (Gibson et al., 2010). Breast milk is the best nutrition for infant growth and development, and the microbiota of breast milk is a factor that significantly influences infant health (Quigley et al., 2013). Probiotics are an important concept for healthcare in the 21st century, and lactic acid bacteria (LAB) are the main source of probiotics (Zielińska and Kolożyn-Krajewska, 2018). LAB are considered to be safe strains and can be used directly. They can regulate the intestinal microbiota, improve the intestinal barrier (Yang et al., 2015), enhance immunity, slow the progression of cancer (Riaz Rajoka et al., 2017), prevent diarrhea (Sirichokchatchawan et al., 2018), and produce bioactive compounds with desirable biological effects (Yerlikaya, 2019).

This study aimed to investigate the effect of phytic acid-degrading bacteria isolated from sow milk on the mineral elements content in mice. Based on a control group with and without 1% phytic acid, the minerals in the liver, kidney, and plasma of mice were determined using an inductively coupled plasma emission spectrometer. The results showed that higher levels of trace element were found in the organs of mice fed with *Lactobacillus lactis* psm16 or commercial phytase in the presence of anti-nutritional factor phytic acid.

## Materials and Methods

### Isolation and Genotypic Identification of Lactic Acid Bacteria Strains

All sow milk samples were serially diluted in sterile phosphate-buffered saline (PBS) and inoculated anaerobically in De Man, Rogosa, and Sharpe (MRS) agar (Hopebio, Qingdao, China) at 37°C for 48–72 h (Xu and Kim, 2014). White colonies were picked and cultured in MRS broth at 37°C for 24 h (Ji et al., 2015). The crystal violet-stained colonies were observed under a microscope using the ×100 oil immersion objective lens. Gram-positive bacteria retained the color of crystalline violet staining, while Gram-negative bacteria lost the initial staining color of neutral red. Subsequently, strains were frozen at −80°C and stored in MRS broth with 50% glycerol until use.

The isolated strains were cultured in MRS broth at 37°C for 18 h under anaerobic conditions and identified *via* the analysis of their 16S rRNA gene sequences. Bacterial DNA was extracted using QIAamp DNA Stool Kit (Qiagen, Gaithersburg, MD, United States) according to the manufacturer’s protocols. The partial 16S rRNA genes was amplified by polymerase chain reaction (PCR) using universal primers 27F (5′-AGAGTTTGATCMTGGCTCAG-3′) and 1492R (5′-GGTTACCTTGTTACGACTT-3′). Reaction was carried out in a reaction volume of 50 μl, containing 25 μl of PrimeSTAR Max Premix 2× (TaKaRa, Shiga, Japan), 16.5 μl of ddH_2_O, 0.5 μmol/l of each primer, and 50–100 ng of DNA template (Rahmdel et al., 2019). PCR was performed under the following conditions: initial denaturation at 96°C for 2 min, followed by 35 cycles of denaturation at 96°C for 10 s, annealing at 56°C for 15 s, and polymerization at 72°C for 1 min. Subsequently, the final polymerization was performed at 72°C for 10 min. PCR products obtained were sequenced by Sanger company (Sangon Biotechnology Co., Ltd., Shanghai, China).

The obtained 16S rRNA sequences were manually corrected and then subjected to Basic Local Alignment Search Tool Nucleotide (BLASTN) analysis to check the similarity with those already deposited sequences in the National Center for Biotechnology Information (NCBI). The retrieved 16S rRNA sequences were aligned using the ClustalW program. The phylogenetic tree of the aligned sequences was performed using the neighbor-joining (NJ) method in MEGA 7 software (Xu et al., 2019).

### Screening of Phytic Acid-Degrading Bacteria

The isolated LAB strains were cultured for 24 h in modified MRS (MRS-MOPS) broth, in which inorganic phosphate (KH_2_PO_4_) was replaced by 0.65 g/l sodium phytate and 0.1 M3-[N-morpholino]propane sulfonic acid (Sangon Biotechnology Co., Ltd., Shanghai, China) (Raghavendra and Halami, 2009). The growth of bacteria in liquid culture media is commonly evaluated by measuring the optical density at 600 nm (OD600) by UV spectrophotometry. Colony-forming units (CFU) were determined by plate counting after gradient dilution (Simova et al., 2008). The bacterial suspension (8 μl of 10^7_^10^8^ CFU/ml) was spotted on a modified MRS (mMRS) agar surface and incubated at 37°C for 24–72 h. Inorganic phosphate (KH_2_PO_4_) in MRS agar medium was replaced by 20 g/l MOPS, 2 g/l calcium chloride, and 2.5 g/l sodium phytate (Fischer et al., 2014). Anhydrous calcium chloride and sodium phytate were filtered through a 0.2-micron filter and added to the autoclaved medium at 55°C. Since the medium was opaque after phytic acid precipitation, the formation of a transparent zone was an indicator of phytic acid hydrolysis.

To avoid false-positive results due to hyaline circles caused by acid solubilization, the colonies on the plates were washed from the agar surface with double-distilled water after incubation and then soaked in 2% (w/v) aqueous cobalt chloride solution for 5 min. Furthermore, an equal volume of 6.25% (w/v) ammonium molybdate solution and 0.42% (w/v) ammonium metavanadate solution was used to replace cobalt chloride hexahydrate for 5 min incubation at room temperature and then removed to check the hydrolysis plate (Bhagat et al., 2020).

### Phytase Enzyme Assay

To further identify the phytase activity of the LAB, preliminarily screened strains were cultured in different centrifuge tubes containing 1 ml MRS broth at 37°C and 900 rpm for 24 h. Then, 50 μl of culture was transferred to 10 ml MRS broth and incubated at 37°C and 200 rpm for 72 h. The culture broth was frozen overnight at −80°C, then concentrated three times in an Ultra-Low Temperature Freezer and centrifuged at 4°C and 8,000 rpm for 10 min. The supernatant was taken and tested for phytase activity analysis with a phytase assay kit (Suzhou Comin Biotechnology Co., Ltd., Suzhou, China). Commercial phytase was directly added to MRS broth as a control. Strains with the highest phytase activity were selected for further study.

### Animals and Experimental Treatments

Four-week-old male Kunming mice (initial body weight 18–21 g) were purchased from Hunan SJA Laboratory Animal Co., Ltd. (Changsha, Hunan, China). The mice were placed under controlled environmental conditions (temperature 22 ± 2°C, relative humidity 55 ± 2%) with light/dark cycles of 12 h. All animals were acclimatized for 3 days prior to an experiment and had free access to food and water. Standard commercially available mouse food (Jiangsu Xietong Pharmaceutical Bioengineering Co., Ltd., Jiangsu, China) was fed separately, and its specific nutritional index is shown (Supplementary Table 1). The experimental protocol was reviewed and approved by the Animal Care and Use Committee (SYXK 2014-0007) of Hunan Normal University. All experiments were performed in accordance with relevant guidelines and regulations.

Thirty-two mice were randomly divided into four groups (Supplementary Table 2), with eight mice per group, and fed different diets for 28 days: (1) control, (2) PA, (3) PA + psm16, and (4) PA + phytase. Group 1 mice were fed the basal diet. Group 2–3 mice received 1% phytic acid supplementation in the basal diet. Group 4 mice were fed a basal diet with 1% phytate and 500 U/kg commercial phytase. Group 1, 2, and 4 mice were gavaged with 200 μl PBS, and group 3 mice were gavaged with strain psm16 (1 × 10^9^ CFU in 200 μl PBS). All groups of mice were gavaged once a day for the first 18 days and then once every 3 days for a total of 21 times. Animals were fasted for 12 h and sacrificed prior to sampling.

Blood samples were placed in plastic tubes containing heparin for at least 3 h and then centrifuged at 4,000 rpm for 15 min at 4°C. The serum was recovered and stored at −80°C to be used for mineral content determination. After blood sampling, the livers, kidneys, and ceca were taken and weighed and stored at −80°C until use. The cecum wall was flushed and weighed, and the cecum contents were used in a short-chain fatty acid (SCFA) assay and pH determination. Feces were collected for an SCFA assay during the last 3 days of the experiment.

### Short-Chain Fatty Acid and pH Analysis

The SCFA of concentration was measured using the method of gas chromatography, as reported earlier with minor modifications (Zhou et al., 2019). Stool and cecum content samples were homogenized and diluted with distilled water. After mechanical vibration and centrifugation at 15,000 rpm for 15 min at 4°C, a mixture of supernatant fluid and 25% metaphosphoric acid solution (9:1) was allowed to stand for 3 h. Then, the mixed solution was centrifuged at 15,000 rpm for 10 min and filtered through a membrane filter (pore size 0.45 μm). SCFAs were measured with a gas chromatography (Agilent Technologies 7890B System) equipped with a DB-FFAP column (30 m × 250 μm × 0.25 μm). The carrier gas was nitrogen (flow 0.8 ml/min, split ratio 50:1, volume of sampling 1 μl). The oven, detector, and injector temperatures were 220, 280, and 250°C, respectively. The SCFA content was quantified using an external standard curve method with standard solutions of acetate, propionate, butyrate, isobutyrate, isovalerate, and valerate (Wang et al., 2017).

The pH of the cecum contents was measured by centrifugation at 8,000 rpm for 1 min at 4°C, then testing 0.8 μl of supernatant with pH paper (pH 5–9) and observing the color change.

### Measurement of Minerals

The levels of copper, iron, zinc, manganese, magnesium, phosphorus, and calcium in mouse liver, kidney, and plasma were determined using an inductively coupled plasma emission spectrometer (ICP-OES 5110, Agilent, Santa Clara, CA, United States) (Wan et al., 2018). The samples were completely dried at 60°C and ground into powder before pretreatment, and approximately 0.2 g frozen tissue and approximately 0.2 ml plasma sample were used in each experiment. Livers and kidneys were decolorized with 1 ml hydrogen peroxide, digested with nitric acid and perchloric acid at 180°C for 100 min, then dried at 260°C, redissolved in 1% HNO_3_, filtered, and subjected to an ICP analysis. The detection wavelengths were Ca (λ = 317.933 nm), Cu (λ = 324.754 nm), Fe (λ = 238.204 nm), Mg (λ = 279.553 nm), Mn (λ = 257.610 nm), P (λ = 213.618 nm), and Zn (λ = 206.200 nm). The metal and trace element content data in the liver, serum, and kidney were expressed as ppm. The content of trace elements in different dietary groups was assayed by the company SGS-CSTC Standards Technical Services Co., Ltd. (Shanghai, China) (Supplementary Table 3).

### Statistical Analysis

Experimental data were initially compiled using the WPS Education Test Edition and then analyzed using IBM SPSS Statistics 20.0. The normality and homoscedasticity of the data were tested before performing a one-way ANOVA. Non-parametric tests were used to ensure the accuracy of the analysis for data that did not meet the assumption of normality and homogeneity of variance, and the Duncan and LSD methods were used to meet the requirements. Phytase data were processed with GraphPad Prism 8 (GraphPad Software, San Diego, CA, United States). The results were expressed as the mean ± standard error, and significance was defined as a *p*-value < 0.05.

## Results

### Screening and Identification of Phytic Acid-Degrading Lactic Acid Bacteria

**FIGURE 1:**
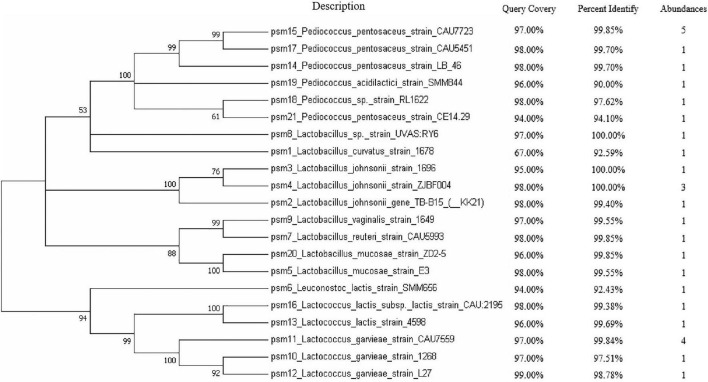
The evolutionary history was inferred using the neighbor-joining method. A bootstrap consensus tree inferred from 1,000 replicates was used to represent the evolutionary history of the taxa analyzed. Branches corresponding to partitions reproduced in less than 50% bootstrap replicates are collapsed. The percentage of replicate trees in which the associated taxa clustered together in the bootstrap test (1,000 replicates) are shown next to the branches whereas the BLAST alignment results are shown. The abundances of the isolated bacterial are shown in the last column.

After cultivation of microbes from sow milk samples, a total of 30 strains were isolated based on colony morphology and 16Sr RNA gene sequence to include as much diversity as possible at the species level. A phylogenetic tree was constructed using an NJ analysis with MEGA 7, which indicated the relative phylogeny of the isolates in comparison to reference strains. *Lactobacillus* (11/30), *Pediococcus pentosaceus* (9/30), and *Lactococcus* (8/30) were the most abundant species in the isolated microbial flora. Minor populations of other species such as *Pediococcus acidilactici* (1/30) and *Leuconostoc lactis* are (1/30) were also seen in the phylogenetic tree (Figure 1).

**FIGURE 2:**
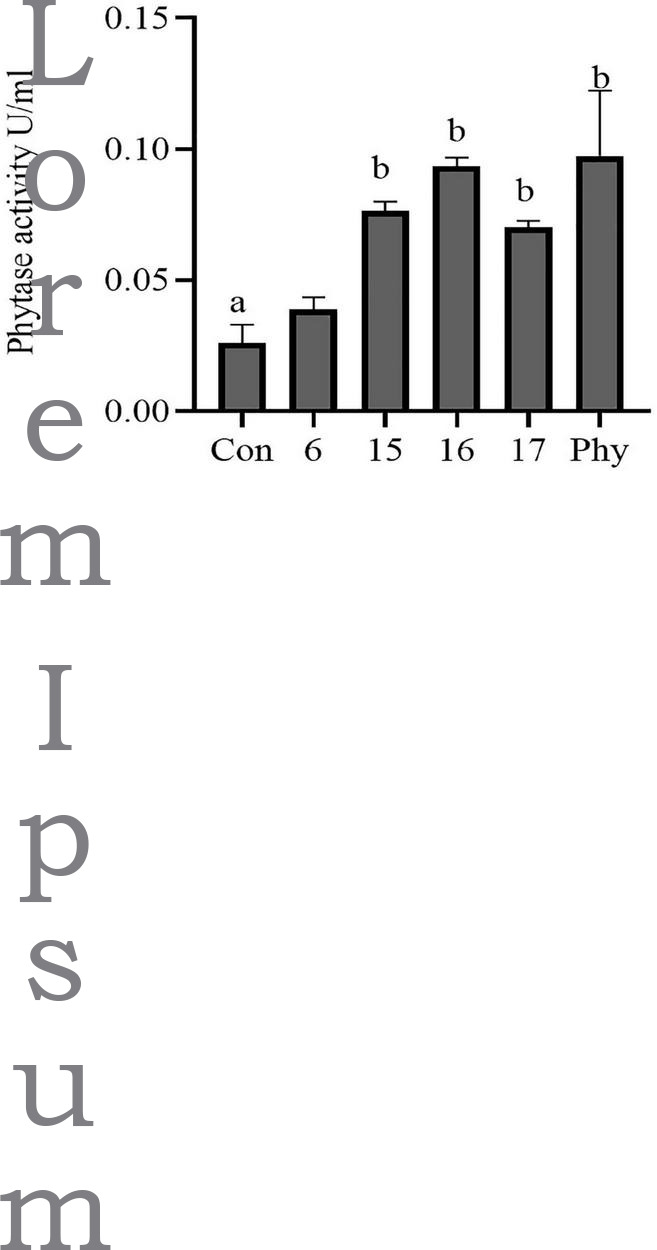
Phytase activity in four selected phytate-degrading strains was assayed. The supernatant of the cultured strains was assayed for phytase activity using a phytase assay kit. Con, MRS; 6, psm 6; 15, psm 15; 16, psm16; 17, psm17; Phy, Phytase. Differences in the bar are not significant without marked letters (*p* > 0.05). a, b values that do not share a common letter in the bar graph above differ significantly (*p* < 0.05).

The isolates were screened for extracellular phytase production with a plate screening method. Psm6, psm15, psm16, and psm17 showed the ability to degrade phytic acid. Psm15 and psm17 are *P. pentosaceus* strains, psm16 is a *Lactococcus lactis* subsp. *lactis* strain, and psm6 is a *L. lactis* strain. Of the four isolates, strain psm16 showed the largest degradation transparent circle on the plate (Supplementary Figure 1) and had the strongest phytase activity (Figure 2). For further verification, microscopic observation indicated that isolated strain psm16 was cocci (Supplementary Figure 2). Strain psm16 was deposited in the China General Microbiological Culture Collection Center (CGMCC 22933).

### Effects of Dietary Conditions on Physiological Variables

The weights of liver, kidney, cecum, and cecum contents and the lengths of large intestine (LIL) and small intestine (SIL) in mice were measured and compared with the animal’s final weight (Table 1). The addition of phytic acid to the diet as well as the gavaged strain had no effect on the final weight of the mice. There were no significant differences in LIL, SIL, liver weight, kidney weight, and cecum weight due to the dietary treatments in mice. In contrast, the addition of strain psm16 significantly increased the weight of the cecum contents (*p* < 0.045), and the addition of commercial phytase had a profound effect on the cecum wall weight (*p* < 0.010). Compared with the basic group, the pH value in the cecum was relatively lower in the phytic acid group and the addition of psm16 restored the pH to the value of the control group and was acidic, but the pH value became slightly alkaline after the addition of phytase (Supplementary Figure 3).

**TABLE 1:** The effects of dietary supplementation with phytic acid and bacterial strains on growth parameters in mice (mean ± SEM).

	Dietary treatment				
Items	BD (*n* = 8)	PA (*n* = 8)	PA + psm16 (*n* = 7)	Phy (*n* = 8)	*p*-value
BW (g)	39.585 ± 0.924	39.740 ± 0.856	38.523 ± 1.301	40.693 ± 1.003	0.54
LW/BW (g/g)	0.036 ± 0.003	0.039 ± 0.003	0.042 ± 0.001	0.038 ± 0.001	0.478
KW/BW (g/g)	0.016 ± 0.000	0.017 ± 0.000	0.017 ± 0.001	0.016 ± 0.001	0.153
LIL/BW (cm/g)	0.216 ± 0.011	0.235 ± 0.016	0.223 ± 0.021	0.229 ± 0.011	0.83
SIL/BW (cm/g)	1.276 ± 0.025	1.293 ± 0.035	1.353 ± 0.039	1.333 ± 0.028	0.321
CW/BW (mg/g)	8.300 ± 0.370	7.700 ± 0.470	8.700 ± 0.450	7.500 ± 0.580	0.346
CWW/BW (mg/g)	5.350 ± 0.326^a^	5.180 ± 0.222^ab^	4.300 ± 0.244^bc^	4.010 ± 0.388^c^	0.010
CCW/BW (mg/g)	2.960 ± 0.459^b^	2.530 ± 0.415^b^	4.360 ± 0.354^a^	3.540 ± 0.509^ab^	0.045

*BD, basal diet; PA, basal diet added with 1% phytic acid; PA + psm16, 1% phytic acid group was treated with Lactococcus lactis psm16; Phy, phytase; BW, body weight; KW, kidney weight; LIL, large intestine length; SIL, small intestine length; CW, cecum weight; CWW, cecal wall weight; CCW, cecal content weight.*

*Differences in the same row are not significant without marked letters (p > 0.05).*

*^a–c^Values in the same row not sharing a common superscript differ significantly (p < 0.05).*

### Effects of Dietary Treatments on Cecal and Fecal Short-Chain Fatty Acid

We investigated whether the various diets influenced SCFA concentrations in mice. Compared to the control, there were changes in the cecal and fecal SCFA levels in mice fed with phytic acid (Table 2). Propionate, butyrate, isovalerate, valerate, and total SCFA were lower in the ceca of mice on phytic acid diets than on basal diet. In addition, acetic acid was significantly reduced. When mice were orally gavaged with strain psm16, the concentrations of acetate (*p* < 0.015), propionate (*p* < 0.014), butyrate (*p* < 0.036), and total SCFA (*p* < 0.009) in the ceca of mice were significantly increased. Compared with the group fed phytic acid alone, the groups fed phytic acid and treated with strain psm16 had higher fecal acetate (*p* < 0.034), propionate (*p* < 0.000), butyrate (*p* < 0.000), and total SCFA (*p* < 0.009) levels. Additionally, significant increases in the acetate and total SCFA levels were observed in the phytase group.

**TABLE 2:** Effects of strains and PA on the levels of SCFA in the cecum and feces (mean ± SEM, *n* = 7).

Items	Dietary treatment				
	BD	PA	PA + psm16	Phy	*p*-value
**Acetate (mg/g)**					
Cecal contents	3.52 ± 0.29^ab^	2.81 ± 0.17^b^	4.30 ± 0.34^a^	3.78 ± 0.36^a^	0.015
Late fecal	2.9 ± 0.24^a^	3.02 ± 0.15^a^	3.6 ± 0.18^b^	3.58 ± 0.14^b^	0.034
**Propionate (mg/g)**					
Cecal contents	0.72 ± 0.07^b^	0.62 ± 0.02^b^	0.90 ± 0.06^a^	0.78 ± 0.06^ab^	0.014
Late fecal	0.52 ± 0.04^a^	0.62 ± 0.03^b^	0.73 ± 0.03^c^	0.75 ± 0.03^c^	0
**Isobutyrate (mg/g)**					
Cecal contents	0.15 ± 0.02	0.12 ± 0.01	0.16 ± 0.01	0.14 ± 0.01	0.08
Late fecal	0.06 ± 0.01^a^	0.08 ± 0.00^ab^	0.08 ± 0.00^ab^	0.09 ± 0.00^b^	0.025
**Butyrate (mg/g)**					
Cecal contents	0.72 ± 0.12^a^	0.40 ± 0.04^b^	0.62 ± 0.06^a^	0.46 ± 0.06^ab^	0.036
Late fecal	0.35 ± 0.05^a^	0.47 ± 0.03^b^	0.63 ± 0.04^c^	0.58 ± 0.03^bc^	0
**Isovalerate (mg/g)**					
Cecal contents	0.16 ± 0.01	0.14 ± 0.01	0.18 ± 0.01	0.16 ± 0.01	0.14
Late fecal	0.09 ± 0.01^a^	0.10 ± 0.01^ab^	0.12 ± 0.01^bc^	0.13 ± 0.00^c^	0.003
**Valerate (mg/g)**					
Cecal contents	0.19 ± 0.02	0.14 ± 0.01	0.19 ± 0.02	0.16 ± 0.02	0.103
Late fecal	0.09 ± 0.01^a^	0.09 ± 0.00^a^	0.11 ± 0.00^ab^	0.12 ± 0.00^b^	0.003
**Total SCFA (mg/g)**					
Cecal contents	5.18 ± 0.47^ab^	4.22 ± 0.18^b^	6.35 ± 0.45^a^	5.49 ± 0.49^a^	0.009
Late fecal	4.01 ± 0.34^a^	4.38 ± 0.22^a^	5.26 ± 0.26^b^	5.24 ± 0.20^b^	0.009

*BD, basal diet; PA, basal diet added with 1% phytic acid; PA + psm16, 1% phytic acid group was treated with Lactococcus lactis psm16; Phy, phytase; SCFA, short chain fatty acids.*

*Differences in the same row are not significant without marked letters (p > 0.05).*

*^a–c^Values in the same row not sharing a common superscript differ significantly (p < 0.05).*

### Effects of Dietary Condition on Trace Elements Status

To verify whether strain psm16 affects the content of mineral elements in mice, we studied the calcium, magnesium, copper, zinc, iron, phosphorus, and manganese levels in the liver, kidney, and plasma. Mineral elements in the various diets were determined, and no differences were evident (Supplementary Table 3). In mice fed with phytic acid, the levels of Fe (*p* < 0.048) and Zn in the liver were lower than in the basal diet group, whereas Cu (*p* < 0.021) and Zn (*p* < 0.017) were significantly higher when strain psm16 was given orally, and Fe was increased in the phytase group (Table 3). In kidney tissue, phytic acid significantly decreased the levels of Fe (*p* < 0.002) and Mn (*p* < 0.010). Gavage with strain psm16 also changed the mineral level in the kidney: a significant increase of Mn and Zn (*p* < 0.022) was seen, whereas the levels of Cu (*p* < 0.007) and Mg (*p* < 0.001) were significantly decreased. Addition of phytase together with a phytic acid diet increased the content of Mn (*p* < 0.010) and decreased the contents of Cu (*p* < 0.007) and Mg (*p* < 0.001) in the kidney (Table 3). The levels of Ca (*p* < 0.000), Mn (*p* < 0.000), and Zn (*p* < 0.000) in plasma were significantly increased by gavage with strain psm16. Cu (*p* < 0.040) was significantly increased in the phytase group compared with the basal diet group (Table 3). In addition, the content of P in the kidney and the plasma was lower after adding phytic acid, but it returned to normal levels after treatment with strain psm16 or phytase, although the differences were not statistically significant.

**TABLE 3:** Effects of strains and phytic acid on the levels of trace elements in the liver, kidney, and plasma (mean ± SEM).

	Dietary treatment				
Items	BD (*n* = 8)	PA (*n* = 8)	PA + psm16 (*n* = 7)	Phy (*n* = 8)	*p*-value
**Liver**					
Ca (ppm)	391.75 ± 28.69	363.91 ± 22.42	360.73 ± 66.06	417.31 ± 57.25	0.469
Cu (ppm)	17.81 ± 0.76^b^	18.27 ± 0.57^b^	21.34 ± 0.51^a^	20.69 ± 1.22^ab^	0.021
Fe (ppm)	316.21 ± 19.05^b^	229.04 ± 13.21^a^	270.25 ± 25.02^ab^	297.10 ± 25.75^b^	0.048
Mg (ppm)	702.84 ± 22.3	724.49 ± 15.86	746.62 ± 19.63	692.44 ± 17.49	0.224
Mn (ppm)	3.31 ± 0.15	3.18 ± 0.16	3.43 ± 0.18	3.34 ± 0.14	0.743
P (ppm)	10689.51 ± 397.21	11050.66 ± 241.84	11465.32 ± 244.71	10752.23 ± 319.96	0.319
Zn (ppm)	133.41 ± 12.90^b^	103.18 ± 3.21^a^	117.11 ± 2.46^b^	111.32 ± 2.57^ab^	0.017
**Kidney**					
Ca (ppm)	736.84 ± 102.78	749.42 ± 37.43	576.76 ± 86.23	684.66 ± 26.38	0.33
Cu (ppm)	27.11 ± 1.94^ab^	27.01 ± 0.66^a^	22.41 ± 2.18^b^	18.88 ± 0.35^b^	0.007
Fe (ppm)	284.98 ± 10.19^a^	237.55 ± 7.08^b^	253.12 ± 4.94^b^	247.08 ± 8.37^b^	0.002
Mg (ppm)	823.86 ± 9.33^a^	829.73 ± 7.15^a^	783.40 ± 8.01^b^	791.47 ± 7.19^b^	0.001
Mn (ppm)	6.14 ± 0.26^b^	4.98 ± 0.22^a^	5.85 ± 0.26^b^	6.09 ± 0.27^b^	0.010
P (ppm)	13702.29 ± 129.12	13458.39 ± 82.07	13780.06 ± 86.35	13785.03 ± 84.47	0.083
Zn (ppm)	88.62 ± 1.26^c^	90.18 ± 2.22^ac^	97.13 ± 4.08^b^	93.39 ± 0.63^ab^	0.022
**Plasma**					
Ca (ppm)	65.28 ± 16.40^a^	61.83 ± 12.29^a^	486.34 ± 177.17^b^	818.32 ± 238.63^b^	0.00
Cu (ppm)	0.62 ± 0.02^a^	0.68 ± 0.03^ab^	0.67 ± 0.06^ab^	0.74 ± 0.02^b^	0.04
Fe (ppm)	3.64 ± 0.35	4.06 ± 0.52	4.84 ± 0.47	3.54 ± 0.35	0.13
Mg (ppm)	22.19 ± 0.61	20.98 ± 0.47	24.55 ± 1.65	23.43 ± 1.39	0.29
Mn (ppm)	37.19 ± 6.27^b^	45.66 ± 4.11^b^	176.34 ± 51.65^a^	283.52 ± 69.37^a^	0.00
P (ppm)	161.45 ± 4.70	156.40 ± 4.03	166.55 ± 9.34	165.30 ± 6.62	0.67
Zn (ppm)	1.57 ± 0.82^a^	1.27 ± 0.71^a^	28.94 ± 11.10^b^	48.70 ± 14.75^bc^	0.00

*BD, basal diet; PA, basal diet added with 1% phytic acid; PA + psm16, 1% phytic acid group was treated with Lactococcus lactis psm16; PHY, phytase.*

*Differences in the same row are not significant without marked letters (p > 0.05).*

*^a–c^Values in the same row not sharing a common superscript differ significantly (p < 0.05).*

## Discussion

Phytic acid is known for its anti-nutritive effects, such as the inhibition of mineral uptake, especially of Cu, Zn, or Fe absorption in the gastrointestinal tract (Lopez et al., 2000). Phytic acid also reduces the activity of enzymes such as trypsin, pepsin, and β-galactosidase (Mohammadi-Kouchesfahani et al., 2019). Moreover, it has been shown that diets high in phytic acid reduce the expression of some genes responsible for appetite and glucose or sodium absorption in fish and piglets, respectively (Woyengo et al., 2012; Liu et al., 2014). Here, in this study we isolated a strain of *L. lactis* psm16 from porcine milk that exhibited better phytate degrading activity. In mice gavaged with strain psm16, a higher content of mineral elements was found in the organs of mice. It is possible that phytase production facilitates phytic acid hydrolysis, thus altering the absorption of host minerals. This is something we need to explore further in the future.

Humans and mice rely on bacteria to break down indigestible dietary ingredients such as phytic acid (Raghavendra and Halami, 2009). Previous studies have shown that SCFAs are bacterial fermentation products, which can promote the digestion and absorption of nutrients, improve intestinal health and immunity, maintain intestinal function, promote mineral absorption and utilization, and improve the structure of the intestinal flora (Scholz-Ahrens et al., 2007). An SCFA assay showed that when compared with the phytic acid group, acetic acid, propionic acid, butyric acid, and total SCFA in the cecum contents and feces increased significantly in the strain psm16 group. SCFAs produced by bacteria are associated with host health, whereas butyrate in feces is significantly associated with an improved insulin response (Serino, 2019).

Zinc is an essential trace element for normal cell function. It is involved in the regulation of enzyme formation, cell signaling, antioxidant defenses, insulin biosynthesis, secretion, and mRNA expression (Maret, 2013). The present study revealed that the Zn content in the phytic acid group was significantly lower than that in the control group in the liver and significantly lower in the plasma than that in the phytase group. In mice gavaged with strain psm16, the Zn content in the liver, kidney, and serum increased significantly. The beneficial effect of strain psm16 on zinc absorption can be explained by at least two possible mechanisms: phytic acid hydrolysis induced by microbial phytase produced by strain psm16 or the existence of organic acids such as lactic acid and SCFA that form soluble ligands with zinc, which prevents the formation of insoluble zinc phytate (Lopez et al., 2000).

Iron is an important element in hemoglobin and myoglobin and plays a vital role in the metabolism of humans and animals. It is also a metal contained in many important enzyme active sites, such as catalase, peroxidase, and cytochrome (Silva and Faustino, 2015). During human life, the iron demand in infants during weaning is the highest per unit weight, and diet is insufficient to meet the iron needs. The absorption of non-heme and heme iron in typical weaning porridge, whether ascorbic acid is present or not, improves iron bioavailability (Hallberg et al., 2003). Evidence also indicates that phytic acid inhibits non-heme Fe absorption in humans (Gillooly et al., 1983). Interestingly, only small amounts of phytic acid (5–10 mg phytates) in a meal are sufficient to reduce Fe absorption (Hallberg et al., 1989). In our study, the presence of 1% phytic acid in the diet altered the Fe levels in the liver and kidney. In mice gavaged with strain psm16 or fed commercial phytase, the Fe content approached control levels. LAB themselves may metabolize trace elements in the chyme and indirectly change host mineral elements. It has been reported that mineral elements were increased in colostrum when fermented with LAB (Bartkiene et al., 2018). Studies on the role of iron in a microbial context are scarce. Although Fe absorption and assimilation *in vivo* have been well established, the mechanism of intestinal flora regulation is not completely clear yet.

Manganese can be released by adding xylanase and phytase from wheat, barley, soybean meal, corn, and wheat bran (Yu et al., 2018). Low levels of manganese support growth and reproduction in pigs, while a lack of manganese causes multiple skeletal abnormalities in poultry and cattle (Spears, 2019). Manganese may also delay pork decomposition and improve pork quality by reducing oxidative damage (Sawyer et al., 2007). In our study, the manganese content in the kidney and plasma increased significantly after adding strain psm16 strain or commercial phytase. The addition of strain psm16 may produce phytase, which degrades phytic acid and improves Mn levels, even if a strong anti-correlation between Mn uptake and phytic acid content in food existed.

Copper is an essential element for several enzymes involved in antioxidant responses, such as superoxide dismutase and for ATP production (Dalecki et al., 2017). Over the years, copper has been widely used as a feed additive in pig production in the United States and China. It promotes pig growth and survival after weaning by increasing feed intake and metabolic activity (Shelton et al., 2011; Carpenter et al., 2019). The current results showed that copper is significantly increased in the liver after the addition of strain psm16, while in the kidney, after the addition of psm16 strain or commercial phytase, the level of copper is still in the normal range, but lower than in phytic acid group alone, and magnesium also shows the same change. In contrast to previous studies, the addition of phytic acid in white wheat bread can inhibit partial apparent magnesium absorption in the human body (Bohn et al., 2004). Various reports document the effect of phytase supplementation on copper metabolism in corn-soybean meal diets. It has been reported that vegetable protein with a high phytic acid level in adults inhibited the absorption of iron, zinc, calcium, and manganese but did not inhibit the absorption of copper (Hurrell, 2003). However, it was also found that phytase supplementation in low phosphorus corn-soybean meal diets increased copper retention in chickens (Banks et al., 2004). The above studies indicated that the element absorption was not changed, while others observed an increase in the bioavailability of elements. These apparent inconsistencies can be attributed to two different circumstances: the different supplied doses of phytate and the nature and the amount of the other dietary components (Grases et al., 2001). The antagonistic relationship between zinc and copper in absorption and utilization may also be related to the higher basal level of copper in the diets. The effect of phytase on copper has not been shown, and the exact mechanism should be further studied.

Milk and dairy products are the most important sources of calcium for people living in developed countries, and plant foods are the main sources of calcium in China. It has been reported that only the highest phytic acid supplementation can significantly reduce serum iron concentration, while calcium and magnesium were not changed (Szkudelski, 2005). In this study, the calcium contents in the liver and kidney were not affected. However, after phytic acid was added to the diet, the calcium content decreased compared with that in the basal diet group, and the calcium concentration increased significantly after adding strain psm16 in the plasma. The lactic acid fermentation products produced by the psm16 strain makes the cecum acidic, which provides good conditions for endogenous phytase to have a role in reducing phytic acid, thereby increasing the concentration of soluble Ca (Rosa-Sibakov et al., 2018).

In conclusion, we isolated a strain of *L. lactis* psm16 from porcine milk that exhibited better phytate-degrading activity. In the presence of the anti-nutritional factor phytic acid, high levels of trace elements were found in the plasma, liver, and kidney of mice orally gavaged with strain psm16. Further studies are essential to revealing the mechanism of phytase production and phytic acid degradation by *L. lactis* psm16 *in vivo* and to optimizing the conditions for phytase production by psm16.

## Data Availability Statement

The original contributions presented in the study are included in the article/Supplementary Material, further inquiries can be directed to the corresponding author/s.

## Ethics Statement

The animal study was reviewed and approved by the Institutional Animal Care and Use Committee at Hunan Normal University.

## Author Contributions

JY and HN contributed to conceiving and designing the experiments. DZ wrote the original draft of the manuscript. DZ, YZ, JL, LW, CC, and QH performed the mouse-feeding trial and sample collection. DZ and LW conducted the strain screening and short-chain fatty acid assay. JL, DZ, and YZ contributed to the trace element analysis experiment. JY, HN, and VR reviewed and revised the manuscript. JY obtained the funding and supervised the project. All authors contributed to the article and approved the submitted version.

## Conflict of Interest

The authors declare that the research was conducted in the absence of any commercial or financial relationships that could be construed as a potential conflict of interest.

## Publisher’s Note

All claims expressed in this article are solely those of the authors and do not necessarily represent those of their affiliated organizations, or those of the publisher, the editors and the reviewers. Any product that may be evaluated in this article, or claim that may be made by its manufacturer, is not guaranteed or endorsed by the publisher.

## Supplementary Material

Supplementary Figure 1 Application of four phytic acid-degrading bacteria in psm. Application of four acid-degrading bacteria in modified MRS plate.

Supplementary Figure 2 The microscope micrographs of psm16 strain.

Supplementary Figure 3 The pH values detection of the cecum contents in mice. Samples were centrifuged at 4°C for 8000 rpm for 1 min, and the further 0.8 μL of the supernatant tested with pH indicator paper (pH 5–9) and the color change was observed.
