# Supplementary material for: Effects of Phytic Acid-Degrading Bacteria on Mineral Element Content in Mice
Source: Front Microbiol. 2021 Nov 22;12:753195. doi: 10.3389/fmicb.2021.753195 (PMC8645864; doi:10.3389/fmicb.2021.753195)
Supplement: Supplementary file 1 [file Data_Sheet_1.docx]

**Table S1.** Nutrition index content of basic feed.

| Items | Contents |
| --- | --- |
| Moisture (g/kg) | 94 |
| Coarse ash (g/kg) | 60.9 |
| Crude protein (g/kg) | 191.5 |
| Crude fat (g/kg) | 57.2 |
| Crude fiber (g/kg) | 30.1 |
| Calcium (g/kg) | 11.0 |
| Total phosphorus (g/kg) | 6.6 |

**Table S2.** Group explanation of different dietary treatments

| Groups | Diet composition | Group’s name |
| --- | --- | --- |
| Group Ⅰ | basal diet | BD |
| Group Ⅱ | basal diet + 1% phytic acid | PA |
| Group Ⅲ | basal diet + 1% phytic acid + *Lactococcus lactis* by gavage | PA + psm16 |
| Group Ⅳ | basal diet + 1% phytic acid + commercial phytase(500u/kg) | Phy |

**Table S3.** Mineral element content of feed in different dietary treatment groups.

|  | Dietary element content |  |  |
| --- | --- | --- | --- |
| Items | BD | PA | Phy |
| Fe (mg/kg) | 284 | 267 | 268 |
| Mn (mg/kg) | 128 | 121 | 120 |
| Cu (mg/kg) | 13 | 12 | 15 |
| Zn (mg/kg) | 115 | 108 | 112 |
| Ca (mg/kg) | 1.21 | 1.11 | 1.11 |
| Total phosphorus% | 0.75 | 0.77 | 0.76 |
