# Supplementary figures and images for: Effects of Phytic Acid-Degrading Bacteria on Mineral Element Content in Mice
Source: Front Microbiol. 2021 Nov 22;12:753195. doi: 10.3389/fmicb.2021.753195 (PMC8645864; doi:10.3389/fmicb.2021.753195)

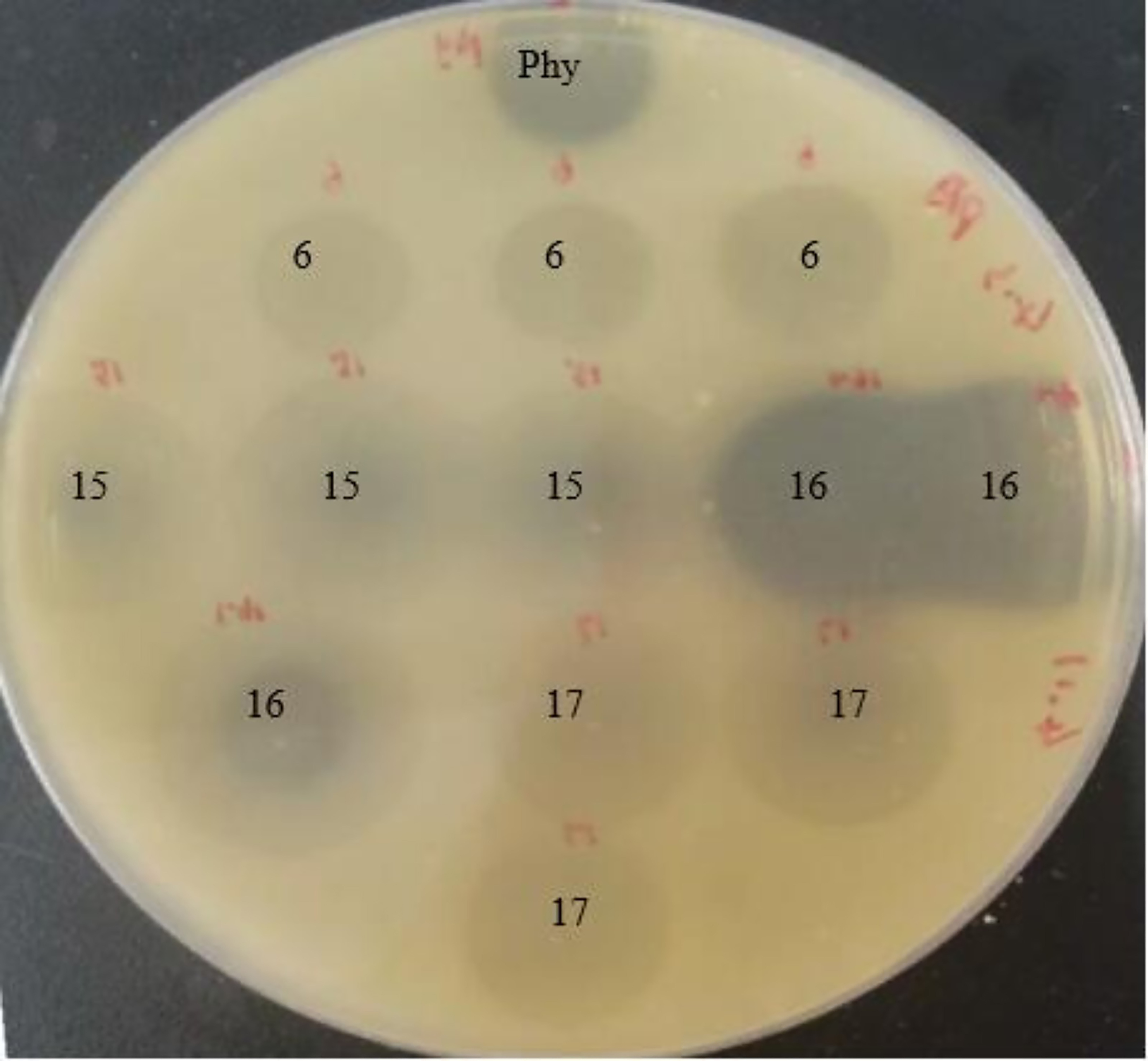

Supplement: Supplementary Figure 1 — Application of four phytic acid-degrading bacteria in psm. Application of four acid-degrading bacteria in modified MRS plate. [file Image_1.jpg]

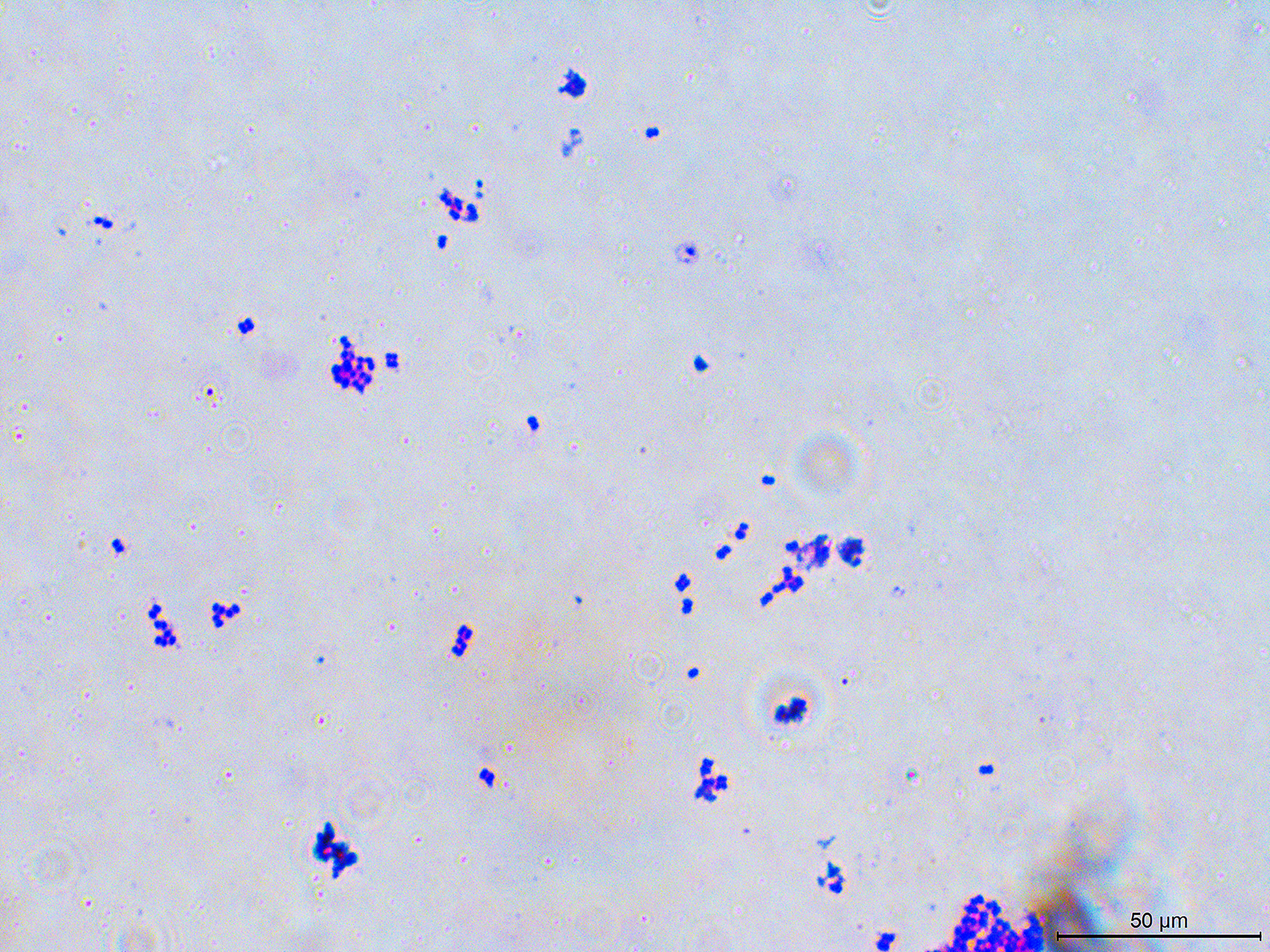

Supplement: Supplementary Figure 2 — The microscope micrographs of psm16 strain. [file Image_2.jpg]

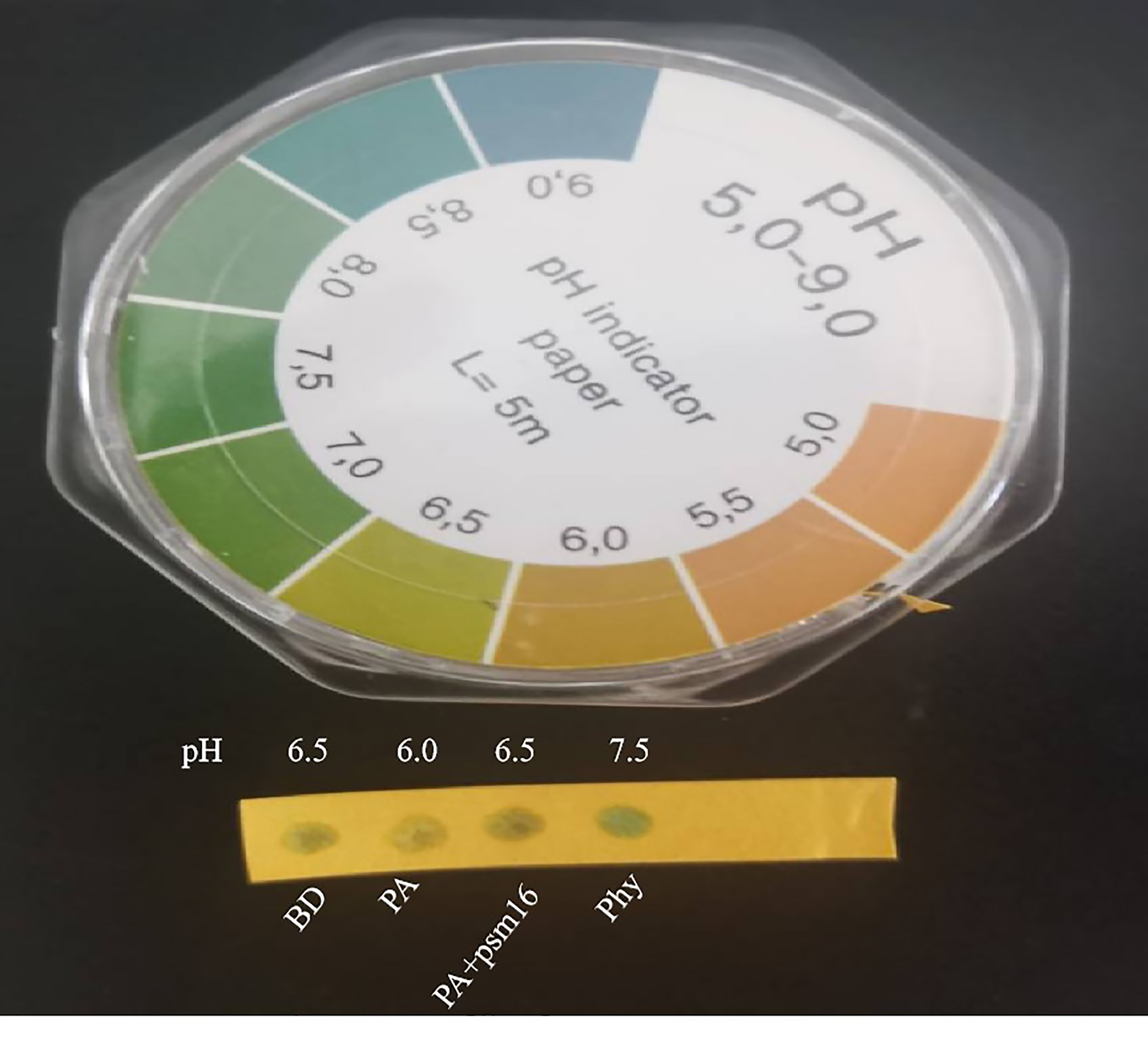

Supplement: Supplementary Figure 3 — The pH values detection of the cecum contents in mice. Samples were centrifuged at 4°C for 8000 rpm for 1 min, and the further 0.8 μL of the supernatant tested with pH indicator paper (pH 5–9) and the color change was observed. [file Image_3.jpg]
